# Strategy for Sensitive and Specific Detection of *Yersinia pestis* in Skeletons of the Black Death Pandemic

**DOI:** 10.1371/journal.pone.0075742

**Published:** 2013-09-17

**Authors:** Lisa Seifert, Michaela Harbeck, Astrid Thomas, Nadja Hoke, Lothar Zöller, Ingrid Wiechmann, Gisela Grupe, Holger C. Scholz, Julia M. Riehm

**Affiliations:** 1 Ludwig Maximilian University of Munich, Department Biology I, Biodiversity research/Anthropology, Martinsried, Germany; 2 State Collection for Anthropology and Palaeoanatomy, Munich, Germany; 3 Bundeswehr Institute of Microbiology, Munich, Germany; 4 Ludwig Maximilian University of Munich, Department of Veterinary Sciences, Institute of Palaeoanatomy, Domestication Research and the History of Veterinary Medicine, Munich, Germany; University of Florence, Italy

## Abstract

*Yersinia pestis* has been identified as the causative agent of the Black Death pandemic in the 14^th^ century. However, retrospective diagnostics in human skeletons after more than 600 years are critical. We describe a strategy following a modern diagnostic algorithm and working under strict ancient DNA regime for the identification of medieval human plague victims. An initial screening and DNA quantification assay detected the *Y. pestis* specific *pla* gene of the high copy number plasmid pPCP1. Results were confirmed by conventional PCR and sequence analysis targeting both *Y. pestis* specific virulence plasmids pPCP1 and pMT1. All assays were meticulously validated according to human clinical diagnostics requirements (ISO 15189) regarding efficiency, sensitivity, specificity, and limit of detection (LOD). Assay specificity was 100% tested on 41 clinically relevant bacteria and 29 *Y. pseudotuberculosis* strains as well as for DNA of 22 *Y. pestis* strains and 30 previously confirmed clinical human plague samples. The optimized LOD was down to 4 gene copies. 29 individuals from three different multiple inhumations were initially assessed as possible victims of the Black Death pandemic. 7 samples (24%) were positive in the pPCP1 specific screening assay. Confirmation through second target pMT1 specific PCR was successful for 4 of the positive individuals (14%). A maximum of 700 and 560 copies per µl aDNA were quantified in two of the samples. Those were positive in all assays including all repetitions, and are candidates for future continuative investigations such as whole genome sequencing. We discuss that all precautions taken here for the work with aDNA are sufficient to prevent external sample contamination and fulfill the criteria of authenticity. With regard to retrospective diagnostics of a human pathogen and the uniqueness of ancient material we strongly recommend using a careful strategy and validated assays as presented in our study.

## Introduction

*Yersinia* (Y.) *pestis* evolved from *Y. pseudotuberculosis* in Central Asia about 1,500–20,000 years ago [1,2]. Since then the agent has spread throughout the world in multiple waves [3]. *Y. pestis* is held responsible for three dreaded pandemics during the history of mankind. First records describe a pandemic wave named after a Roman emperor: Justinian’s plague supposedly lasted from 541 to 750 A.D. [4]. The beginning of the second pandemic became known as the *Black Death* originating from the Latin expression *atra mors*, whereas “*atra*” can be translated with “black” or “terrible” [5]. It was part of the so-called second pandemic, which started in 1345/6 and lasted for several centuries [1,5]. Some historians, however, questioned whether the etiological agent was *Y. pestis*, as in their opinion symptoms and epidemiology of the two early pandemics hardly corresponded to those of the modern plague [6,7]. Since 1894 the most recently evolved biovar Orientalis spread to various countries. Named *modern plague*, it is still endemic in Asia, Africa, and America causing 1,000 to 3,000 noted human cases each year including up to 230 deaths [3].

While the detection of *Y. pestis* in today’s plague victims can be achieved without major difficulties, the detection in ancient samples such as skeletons is crucial. In 1998 *Y. pestis* DNA could be recovered for the first time from 400 year-old skeletons [8]. Since this first description further detection of *Y. pestis* DNA in human remains has been published [8–12]. Just recently the whole genome sequence of *Y. pestis* from a Black Death victim could be determined [13]. And quantitative PCR for the detection of the ancient plague pathogen was successfully described in one prior project [11].

Many criteria of authenticity for the work with aDNA were set up in the past [14–16]. Molecular methods, such as PCR require additional optimization, since ancient DNA (aDNA) quantity, quality, and the level of inhibition are unique to each extract [17]. However, to avoid generating false positive results the performance of PCR as an amplification method must be done carefully and external contamination needs to be excluded. On the contrary, false negative results may also occur if non-validated PCR assays with a low detection limit are used. For instance, Gilbert et al. failed to identify *Y. pestis* specific DNA from five different burial sites using various *Y. pestis* specific primer sets targeting *pla* and *rpoB* [18]. Nguyen-Hieu et al. published a qPCR assay using binding fluorescent probes for the detection of *Y. pestis* and six other pathogens [19]. They were screening more than 1000 samples; however none of them were positive. Controversial results regarding molecular typing assays led to even greater discussion among authors [10,11,20]. It is therefore important to use validated PCR assays to exclude both false positive and false negative results which in consequence may lead to misinterpretation on the presence or absence of a specific pathogen [21].

Although destructive sampling of skeletal remains is permitted, ethical issues relevant for analysis of ancient human remains must also be respected. Destructive procedures should be kept to a minimum in order to preserve valuable material for continuing research such as molecular typing or even whole genome sequencing.

In this study we developed a robust aDNA workflow to detect *Y. pestis* in skeletal remains, consisting of optimized sample preparation in combination with thoroughly validated quantitative screening PCR assays. The assays are easy to perform and allow sensitive and specific detection of *Y. pestis* at a limit of detection (LOD) as low as 4 gene copies. The overall procedure is consistent with the requirements of the international standard ISO 15189 for human diagnostics in accredited laboratories and thus provides a helpful tool in high-quality *Y. pestis* aDNA research.

## Material and Methods

### Ethics Statement

In our study we are analyzing 300-600 year-old archaeological human remains from Bavaria and Brandenburg, Germany, and from Basel, Switzerland.

Two of the coauthors, Prof. Dr. Gisela Grupe and Dr. Michaela Harbeck, are representatives of the State Collection for Anthropology and Palaeoanatomy, Munich, Germany. The State Collection is the responsible authority of the Federal State of Bavaria for conservation of and scientific research on archaeological skeletal remains found in Bavaria, for this study for all samples from Manching-Pichl.

The samples from Brandenburg were provided and investigation was authorized by the official authority “Brandenburg Landesamt für Denkmalpflege und Archäologisches Landesmuseum“. They belong to a government agency of the Federal State of Brandenburg, whose mission is the preservation of the cultural and historical heritage of Germany.

The samples from Basel were provided by the research department “Archäologische Bodenforschung Basel-Stadt”. It is affiliated to the Department of Presidential Affairs of the Canton of Basel-Stadt. Its aim is to protect and care for the canton’s archaeological heritage.

According to the authorities’ rules we did not need further consent or permission to research on the ancient human material.

### Origin of samples

In this study, 29 individuals recovered from three different collectives were investigated (Table 1). The first collective, a mass burial site in Southern Germany, dates to the Gothic period, approximately 1250 to 1500 A.D. [9,22]. Individuals had been recovered from the sacristy of the St. Leonhard Catholic church in Manching-Pichl (MP), near Ingolstadt in Bavaria. Teeth from 20 individuals were tested in this study (Table 1). The second burial site was located in the city of Brandenburg, State of Brandenburg, in the northeast of Germany. Three individuals had been recovered and dated to the Thirty Years’ War at approximately 1640 A.D. [23]. The third collective was the mass burial site Elisabethengottesacker in Basel, Switzerland. This cemetery had been in use from the end of the 13^th^ to the beginning of the 19^th^ century [24]. Six individuals from Basel were analysed in the present study, three of the total have been dated to the 14^th^/15^th^ century by radiocarbon dating (Table 1).

**Table 1:** The investigated ancient samples originated from three different burial sites.

**Burial site**	**Age (A.D.**)	**Individuals tested (positive**)** in this study**	**Positive individual**	**Quantitative screening PCR targeting *pla* (70 bp**)	**Maximum *pla* gene copies per 1µl**	**Specific *pla* amplicons & sequence (133 nt**)	**Specific *caf1* amplicons & sequence (161nt**)
Manching-Pichl, Germany	1250-1500	20 (4)					
			MP17-I	4/4	560	3/3	2/3
			MP19-II	4/4	700	3/3	1/2
			MP59-I	4/4	22	3/3	1/3
			MPS01-I	4/4	3	1/3	0/2
Brandenburg, Germany	1640	3 (3)					
			B1	1/4	≤ 1	0/3	n.d.
			B2	2/4	2	0/3	n.d.
			B3	4/4	6	2/3	1/2
Basel, Switzerland	1300-1490	6 (0)		neg			
		**13 extraction controls**		0/4		0/3	n.d.

Overall results are summarized in Table S1.

PCR assay results are generated from the first DNA extraction round, following the most efficient method [33].

In order to judge the general biomolecular preservation status of the analysed specimens we scrutinized the integrity of the collagenous bone portion. Amino acid analysis was carried out in the facilities of the anthropological work group at the Ludwig Maximilians University (LMU). Three individuals from the mass grave Manching-Pichl were exemplarily investigated: MP 54-II, MP 59-I, and MP 68-I. Collagen extraction, the determination of collagen net yield as well as the assessment of C %, N % and C/N molar ratio were carried out as described elsewhere [25]. 1 mg of the extract was weighed into glass vials and hydrolyzed in 1 ml of 6 N HCl for 14 h at 115 °C. Anoxic conditions were created by flushing the vials with nitrogen. After evaporation of the acid, the hydrolyzate was resuspended in lithium citrate buffer (Sykam Chromatography, Fuerstenfeldbruck, Germany) and diluted for amino acid analysis which was conducted in a Li-HPLC amino acid analyzer with ninhydrin post-column derivatization (S433, Sykam Chromatography, Fuerstenfeldbruck, Germany) for amino and imino acid detection. The chromatograms were calibrated to an amino acid standard (Sykam Chromatography, Fuerstenfeldbruck, Germany) using the software ChromStar 7 (SCPA, Weyhe-Leeste, Germany).

### Concept to Avoid Carryover DNA in PCR

**Figure 1:**
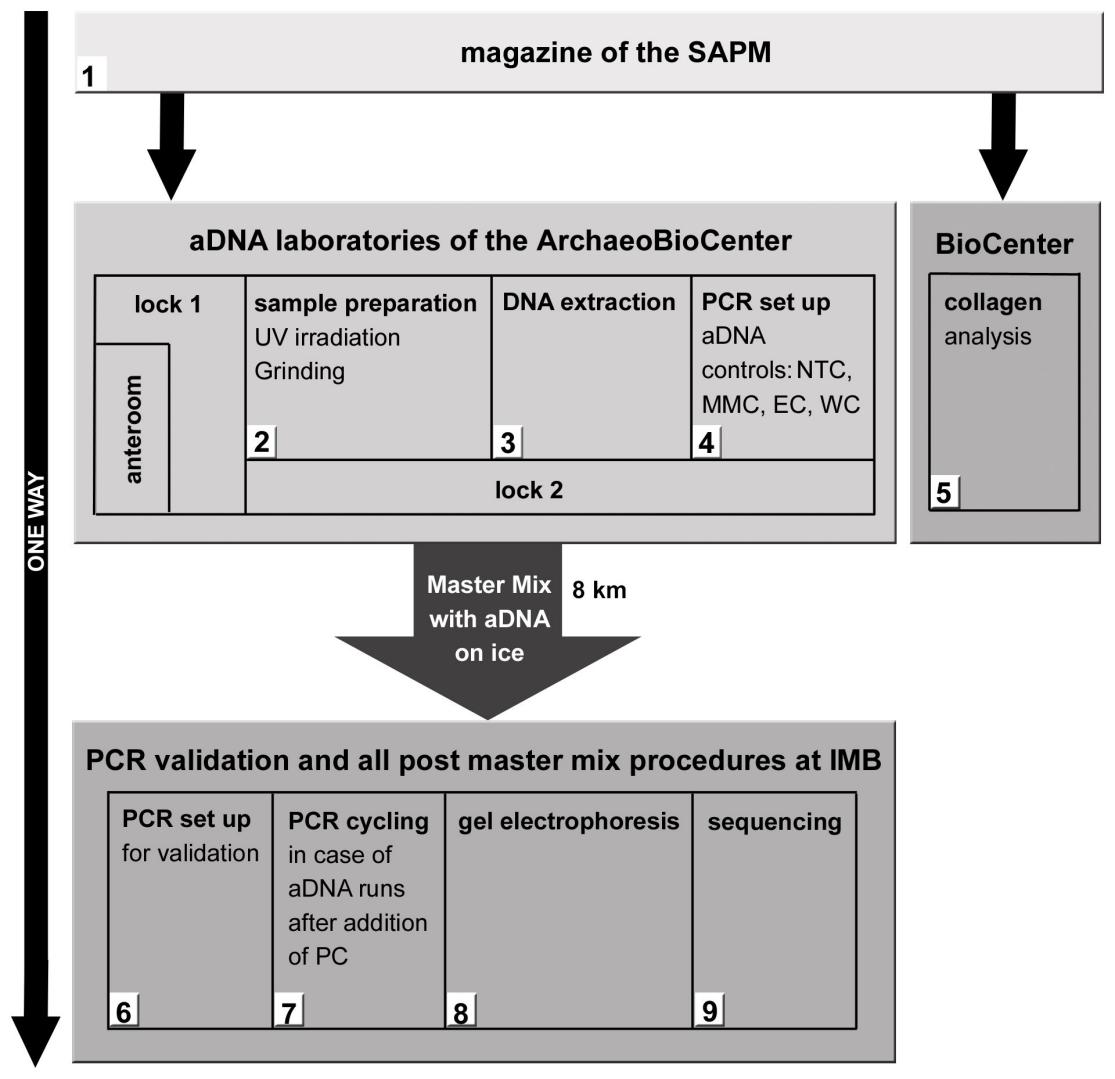
Schematic workflow for the proceeding through highly optimized and validated protocols for the detection of *Y. pestis* from ancient samples.

Sample preparation and ancient DNA (aDNA) extraction were conducted at newly established laboratories of the ArcheoBioCenter of the LMU Munich, Germany. The clean laboratory complex contains three separate rooms, one for each step of the pre-PCR aDNA processing: sample preparation, DNA extraction and PCR setup (Figure 1) [26]. The three rooms are entered through a double air lock system preceding the laboratory. The lab complex is positively pressurized to prevent infiltration of exogenous DNA. All three labs are equipped with UV air cleaners. Without exception, the entire equipment and all consumables were decontaminated with bleach and were UV irradiated prior to introduction into the working areas. Consumables were admitted directly from the producer only. The complete workflow was carried out in a strict one way regime (Figure 1). Staff protocol required personnel to shower, wash hair, and wear freshly laundered clothes prior to entering the lab area. In the gowning room clothing was exchanged to underclothes, and a first pair of gloves and a hairnet were put on (VWR, Ismaning, Germany). In the second room overalls which have a hood to cover the head (DuPont^TM^ Tyvek^®^, Germany), a facemask with screen (Nitritex, Suffolk, United Kingdom), and finally a second pair of gloves were put on. Before entering any of the three aDNA rooms, a second set of overalls was put on covering the body and head.

 Recovered skeletons are housed at the collection depot of the State Collection for Anthropology and Palaeoanatomy (SAPM) (1). Only selected samples were transferred to the aDNA laboratory of the ArchaeoBioCenter (2-4). For PCR setup, reaction tubes were sealed until analysis containing aDNA, no template control (NTC), extraction control (EC), master mix control (MMC), and water control (WC). Further tubes containing master mix were generated for later addition of the positive control (7) at the Bundeswehr Institute of Microbiology (IMB).

PCR reaction tubes containing master mix and aDNA were prepared and sealed in the aDNA lab complex (Figure 1). The reactions were transferred to the Bundeswehr Institute of Microbiology, Munich, Germany (IMB), the second laboratory complex located about 8 km away. The research team is in ISO 15189 compliance and has accreditation for the clinical diagnostics of plague in human samples. At this institution the assay validation was carried out in again a strict one way regime. One laboratory was assigned to master mix preparation, one to the final addition of positive controls and PCR cycling, and a third lab complex housed on a different floor of the building to all post-PCR procedures such as gel analysis as well as DNA sequencing (Figure 1). Concerning the aDNA samples, PCR cycling and any post PCR procedures were carried out at IMB.

The following controls routinely accompanied each round of extraction and were finally analysed along with the aDNA specimens to monitor potential contamination. During the extraction procedure, one blank control (EC) was carried along for every seven samples processed in one round. It contained all reagents except the ancient sample powder. For PCR assays, a master mix control, containing solely master mix (no template control, NTC), and a reaction containing water instead of DNA (water control, WC) were generated as well as a positive control (PC) that contained an artificial DNA construct or DNA from *Y. pestis* strain EV76 (*caf1* assay). Positive controls were added at the IMB location only (Figure 1).

To minimize misleading results due to potentially deaminated cytosine in aDNA we routinely ran each PCR with uracil-DNA glycosylase (UDG). The enzyme cleaves uracil from the aDNA strand originating from deaminated cytosine [27]. If UDG was not included in the master mix provided by the manufacturer, we added the enzyme to each reaction at a concentration according to the manufacturer’s instructions.

### PCR validation

For initial screening of aDNA and quantification of *Y. pestis* specific DNA, a 5’-nuclease assay was set up (qPCR). The 70 base pair (bp) target is located on the plasminogen activator gene (*pla*) [28] within the multi copy plasmid pPCP1 [29]. For confirmation of positive results, a conventional PCR assay, targeting a 133 bp region of the same gene as well as an assay for a second target for the fraction 1 capsule antigen gene (*caf1*) within plasmid pMT1 were validated.

Linear synthetic oligonucleotides as well as *Y. pestis* negative ancient bone matrix were used as controls in assay validation at IMB. Design of primers and probes was carried out using Gene Runner Version 3.05, (Softpedia freeware, http://www.softpedia.com/get/Science-CAD/Gene-Runner.shtml). A special locked nucleic acid probe was designed and produced by TibMolbiol (Berlin, Germany). The resulting PCR products were sequenced and subsequently verified using BLAST search in GenBank (http://blast.ncbi.nlm.nih.gov). To detect PCR inhibitors, a PCR assay targeting the human leukocyte antigen (HLA) gene was modified and validated for the use of aDNA [30].

Specific assay validation was carried out for each assay as described below. Finally probit analyses were carried out to determine the exact limit of detection value (LOD). Quantification and target copy count were possible by generating a standard curve, which was deposited and further used for calculation by the LightCycler 480 II software version 1.5 (Roche, Mannheim, Germany). Assay specificity was tested using 1 ng of DNA per reaction of 41 clinically relevant bacteria, and 29 *Y. pseudotuberculosis* strains as well as DNA of 22 *Y. pestis* strains and 30 previously confirmed clinical human plague samples (Table 2) [31].

**Table 2:** Origin of DNA used as specificity panel, positive and negative samples.

**Species**	**Biovar/biotype**	**Origin**	***pla* qPCR**	**conventional *pla/caf1* PCR**
*Y. pestis*	Pestoides	G8786 Georgia	+	+
*Y. pestis*	Antiqua 1.ANT	Margaret	+	+
*Y. pestis*	Antiqua 1.ANT	CEB87-021 (343)	+	+
*Y. pestis*	Antiqua 1.ANT	NCTC_570 Bombay267	+	+
*Y. pestis*	Antiqua 1.ANT	NCTC_10029 13925/58	+	+
*Y. pestis*	Antiqua 2.ANT	Kuma	+	+
*Y. pestis*	Antiqua 2.ANT	Yokohama	+	+
*Y. pestis*	Antiqua	Kenya 129	+	+
*Y. pestis*	Medievalis	KIM	+	+
*Y. pestis*	Medievalis	Pestis Kurdistan Rodent 24	+	+
*Y. pestis*	Medievalis	Pestis Kurdistan Rodent 28	+	+
*Y. pestis*	Orientalis	EV76, vaccine strain	+	+
*Y. pestis*	Orientalis	NCTC_2028	+	+
*Y. pestis*	Orientalis	NCTC_8775 139L	+	+
*Y. pestis*	Orientalis	CEB02-417, Vietnam	+	+
*Y. pestis*	Orientalis	Java10	+	+
*Y. pestis*	Orientalis	K120-43 87/30	+	+
*Y. pestis*	Orientalis	CEB02-107 (6/69)	+	+
*Y. pestis*	Orientalis	Bara Banki 3	+	+
*Y. pestis*	Orientalis	CEB02-455	+	+
*Y. pestis*	Orientalis	M23	+	+
*Y. pestis*	Orientalis	TS	+	+
*Y. pestis*	Orientalis	Confirmed clinical sample, Madagascar, M401	+	+
*Y. pestis*	Orientalis	Confirmed clinical sample, Madagascar, M402	+	+
*Y. pestis*	Orientalis	Confirmed clinical sample, Madagascar, M403	+	+
*Y. pestis*	Orientalis	Confirmed clinical sample, Madagascar, M404	+	+
*Y. pestis*	Orientalis	Confirmed clinical sample, Madagascar, M406	+	+
*Y. pestis*	Orientalis	Confirmed clinical sample, Madagascar, M407	+	+
*Y. pestis*	Orientalis	Confirmed clinical sample, Madagascar, M408	+	+
*Y. pestis*	Orientalis	Confirmed clinical sample, Madagascar, M409	+	+
*Y. pestis*	Orientalis	Confirmed clinical sample, Madagascar, M410	+	+
*Y. pestis*	Orientalis	Confirmed clinical sample, Madagascar, M411	+	+
*Y. pestis*	Orientalis	Confirmed clinical sample, Madagascar, M412	+	+
*Y. pestis*	Orientalis	Confirmed clinical sample, Madagascar, M413	+	+
*Y. pestis*	Orientalis	Confirmed clinical sample, Madagascar, M414	+	+
*Y. pestis*	Orientalis	Confirmed clinical sample, Madagascar, M415	+	+
*Y. pestis*	Orientalis	Confirmed clinical sample, Madagascar, M418	+	+
*Y. pestis*	Orientalis	Confirmed clinical sample, Madagascar, M421	+	+
*Y. pestis*	Orientalis	Confirmed clinical sample, Madagascar, M422	+	+
*Y. pestis*	Orientalis	Confirmed clinical sample, Madagascar, M423	+	+
*Y. pestis*	Orientalis	Confirmed clinical sample, Madagascar, M424	+	+
*Y. pestis*	Orientalis	Confirmed clinical sample, Madagascar, M425	+	+
*Y. pestis*	Orientalis	Confirmed clinical sample, Madagascar, M426	+	+
*Y. pestis*	Orientalis	Confirmed clinical sample, Madagascar, M427	+	+
*Y. pestis*	Orientalis	Confirmed clinical sample, Madagascar, M429	+	+
*Y. pestis*	Orientalis	Confirmed clinical sample, Madagascar, M431	+	+
*Y. pestis*	Orientalis	Confirmed clinical sample, Madagascar, M432	+	+
*Y. pestis*	Orientalis	Confirmed clinical sample, Madagascar, M433	+	+
*Y. pestis*	Orientalis	Confirmed clinical sample, Madagascar, M435	+	+
*Y. pestis*	Orientalis	Confirmed clinical sample, Madagascar, M437	+	+
*Y. pestis*	Orientalis	Confirmed clinical sample, Madagascar, M438	+	+
*Y. pestis*	Orientalis	Confirmed clinical sample, Madagascar, M439	+	+
*Acinetobacter baumanii*		DSM 7324	-	-
*Bacillus anthracis*		Vollum	-	-
*Bacillus cereus*		ATCC 10987	-	-
*Bacillus* *globigii*		DSM 7264	-	-
*Bacillus* *thuringensis*		DSM 2046	-	-
*Brucella melitensis*		ATCC 23456	-	-
*Burkholderia cepacia*		NCTC 10744	-	-
*Burkholderia mallei*		ATCC_23344	-	-
*Burkholderia pseudomallei*		ATCC_23343	-	-
*Burkholderia* *thailandesis*		DSM 13276	-	-
*Campylobacter jejuni*		ATCC 29482	-	-
*Candida albicans*		ATCC 36232	-	-
*Chlamydophila pneumoniae*		ATCC 53592	-	-
*Citrobacter freundii*		DSM 30039	-	-
*Clostridium perfringens*		ATCC 12915	-	-
*Clostridium* *sporogenes*		DSM 795	-	-
*Coxiella burnetii*		Nine Mile	-	-
*Eikenella* *corrodens*		DSM 8340	-	-
*Enterobacter aerogenes*		DSM 30053	-	-
*Enterococcus faecalis*		DSM 2570	-	-
*Escherichia coli*		ATCC 11303	-	-
*Francisella tularensis*	ssp. *holarctica*	Isolate from patient	-	-
*Haemophilus influenzae*		ATCC 10211	-	-
*Klebsiella pneumoniae*		ATCC 13883	-	-
*Legionella pneumophila*		NCTC 10332	-	-
*Listeria monocytogenes*		DSM 12464	-	-
*Moraxella catarrhalis*		DSM 9143	-	-
*Mycobacterium tuberculosis*		Isolate from patient	-	-
*Neisseria* *meningitidis*		Isolate from patient	-	-
*Propionibacterium acnes*		DSM 1897	-	-
*Proteus mirabilis*		DSM 788	-	-
*Pseudomonas aeruginosa*		ATCC10145	-	-
*Salmonella* *typhi*		Isolate from patient	-	-
*Serratia marcescens*		DSM 1636	-	-
*Shigella dysenteriae*		Isolate from patient	-	-
*Staphylococcus aureus*	toxin B positive	DSM 19041	-	-
*Staphylococcus epidermidis*		DSM 1798	-	-
*Stenotrophomonas maltophilia*		ATCC 5131	-	-
*Streptococcus pneumoniae*		DSM 20566	-	-
*Streptococcus pyogenes*		DSM 20565	-	-
*Vibrio cholerae*		ATCC 15748	-	-
*Y. pseudotuberculosis*		Y003	-	-
*Y. pseudotuberculosis*		Y004	-	-
*Y. pseudotuberculosis*		Y005	-	-
*Y. pseudotuberculosis*		Y076	-	-
*Y. pseudotuberculosis*		Y077	-	-
*Y. pseudotuberculosis*		Y078	-	-
*Y. pseudotuberculosis*		Y080	-	-
*Y. pseudotuberculosis*		Y130	-	-
*Y. pseudotuberculosis*		Y225	-	-
*Y. pseudotuberculosis*		Y227	-	-
*Y. pseudotuberculosis*		Y239	-	-
*Y. pseudotuberculosis*		Y241	-	-
*Y. pseudotuberculosis*		Y248	-	-
*Y. pseudotuberculosis*		Y252	-	-
*Y. pseudotuberculosis*		Y259	-	-
*Y. pseudotuberculosis*		Y260	-	-
*Y. pseudotuberculosis*		Y250	-	-
*Y. pseudotuberculosis*		Y428	-	-
*Y. pseudotuberculosis*		Y711	-	-
*Y. pseudotuberculosis*		Y716	-	-
*Y. pseudotuberculosis*		Y718	-	-
*Y. pseudotuberculosis*		Y719	-	-
*Y. pseudotuberculosis*		Y724	-	-
*Y. pseudotuberculosis*		Y728	-	-
*Y. pseudotuberculosis*		Y731	-	-
*Y. pseudotuberculosis*		Y732	-	-
*Y. pseudotuberculosis*		Y734	-	-
*Y. pseudotuberculosis*		Y735	-	-
*Y. pseudotuberculosis*		Y781	-	-

### Quantitative screening assay targeting *pla*

For all qPCR assays a LightCycler 480 II platform (Roche, Mannheim, Germany) was used. Data analysis and subsequent quantification were performed using the above mentioned LightCycler 480 II software. The 2^nd^ derivative maximum algorithm was determined to calculate the Crossing Point (CP) cycle.

For the detection of *pla* we used *Y. -pest_F* gactgggttcgggcacatg as forward primer, and *Y. -pest_R* cggatgtcttctcacgga as reverse primer. The sequence of the locked nucleic acid probe Y. _pest_TM was 6Fam-tgatgagcacta+tat+g+a+gag-BBQ. LNA probes and primers were purchased from TibMolbiol (Berlin, Germany). Four different master mixes were compared regarding their efficiency: LightCycler^®^ FastStart DNA MasterPLUS HybProbe (Roche, Mannheim, Germany), LC480^®^ Probe Master (Roche, Mannheim, Germany), Platinum^®^ Quantitative PCR SuperMix-UDG with Rox (Invitrogen, Darmstadt, Germany), and LightCycler^®^ TaqMan Master (Roche, Mannheim, Germany). Furthermore various concentrations of magnesium chloride (MgCl_2_), primers and probes were tested. MgCl_2_ concentrations varied from 3 mM to 6 mM in 1 mM steps. Primers were tested at concentrations of 0.2 µM, 0.25 µM, 0.4 µM, 0.5 µM, 0.6 µM, 0.75µM, 0.8 µM and 1 µM. The LNA probe was tested in concentrations of 0.1 µM, 0.125 µM, 0.2µM, 0.25 µM, 0.3 µM, 0.375 µM, and 0.4 µM. To determine the linear range and R^2^ value we used concentrations of the *pla* construct varying from 10,000 copies to zero. We compared qPCR runs with and without bovine serum albumine (BSA, Ambion/ Life Technologies, Darmstadt, Germany) to determine any effect on the assay. The concentrations varied from 0.04 mg/ml to 0.4 mg/ml, 1 mg/ml, 1.5 mg/ml, and in 1 mM steps from 2 mg/ml to 6 mg/ml. The optimal annealing temperature was tested in 2 °C steps from 56 °C to 62 °C.

### Conventional PCR targeting *Y. pestis* specific plasmids and subsequent sequence analysis

Conventional PCR assays amplifying the pPCP1 specific *pla* gene (133 bp) and pMT1 specific *caf1* gene (161 bp) were performed in an Eppendorf Mastercycler Pro (Wesseling-Berzdorf, Germany) instrument. Primers for *pla* specific amplification were the above described *Y. -pest_F*, and the previously published *Y. -pest_R2* AgACTTTggCATTAggTgTg [28]. Primers for *caf1* specific amplification were caf1_F1 aaccagcccgcatcactctta and caf1_R1 atcacccgcggcatctgta [10].

For the *pla* specific assay, six different master mixes or taq polymerases were compared regarding their efficiency: Qiagen Multiplex PCR Master Mix^®^ (Qiagen, Hilden, Germany) with UDG, Qiagen Multiplex PCR plus Master Mix (Qiagen, Hilden, Germany), Platinum^®^ PCR SuperMix High Fidelity (Invitrogen, Karlsruhe, Germany), Qiagen HotStarTaq plus (Qiagen, Hilden, Germany), as well as AmpliTaq^®^ Gold and AmpliTaq^®^ Gold Polymerase LD (both Applied Biosystems/ Life technologies, Darmstadt, Germany). MgCl_2_ was tested in varying concentrations starting with 1.5 mM and from 3 mM to 5 mM in 1 mM steps. To optimize the cycling conditions, the initial denaturation time was altered from 2 min, 5 min, 10 min, to 15 min. The optimal annealing temperature was tested in 2 °C steps from 60 °C to 68 °C using gradient cycling. Moreover, conventional cycling conditions were compared with a touchdown protocol. If runs could be performed with and without UDG both possibilities were tested in order to detect any negative effects of UDG on the result.

Regarding *pla* and *caf1* specific assays, 5 µl of each amplicons were visualized on a 2% agarose gel. Positive samples were purified using the QIAquick PCR Purification Kit (Qiagen, Hilden, Germany) according to the manufacturer’s instructions. For the sequencing reaction we used 1x BigDye terminator v.3.1 Cycle Sequencing Ready reaction Mix (Applied Biosystems, Life technologies, Darmstadt, Germany), 1 pmol/µl of the respective primer and 3-5 µl of purified DNA in a final volume of 10 µl. The reaction was run on a GeneAmp 9700 (Applied Biosystems, Life technologies, Darmstadt, Germany) instrument, starting with an initial denaturation step for 1 min at 96 °C, followed by 25 cycles at 96 °C for 10 sec, 50 °C for 5 sec and 60 °C for 2 min and ending with cooling at 4 °C until further processing. After purification using the Dye Ex 2.0 Spin Kit (Qiagen, Hilden, Germany) sequences were generated on a Genetic Analyzer 3130 (Applied Biosystems, Life technologies, Darmstadt, Germany) instrument. Sequences were analysed using the software Codon Code Aligner V 4.0.4, blasted (http://blast.ncbi.nlm.nih.gov/Blast.cgi) and aligned to the reference sequences GenBank accession AL109969 (pPCP1) and AL117211.1 (pMT1).

### Quantitative PCR assay to determine PCR inhibition

To determine PCR inhibition a 5’-nuclease assay was validated targeting the human leukocyte antigen (HLA) gene (97 bp). Primers and probe were HLA_fwd_2_gaatttgatggagatgagcag, HLA_rev_2_gcgggtcaaaacctccaaat, and HLA_p1 6FAM-TACgTggACCTggAgAggAAggAgACT-BHQ1, respectively. Two different master mixes were compared regarding their efficiency: Platinum^®^ Quantitative PCR SuperMix-UDG with Rox (Invitrogen/ Life technologies, Darmstadt, Germany) and LightCycler^®^ FastStart DNA Master Hybridisation Probes (Roche, Mannheim, Germany). The annealing temperature was tested at 60 °C and 63 °C. Various concentrations of MgCl_2_, primers and probe were used. For MgCl_2_, concentrations of 3 mM to 6 mM were tested in 1 mM steps. Primers were tested at concentrations of 0.2 µM, 0.3 µM, 0.4 µM, and 0.6 µM and the probe at concentrations of 0.2 µM, 0.4 µM, and 0.6 µM, respectively.

Regarding the overall interpretation of PCR outcomes, a positive result was assigned, if the CP value of a qPCR was below 50 (*pla*) and 45 (*HLA*), respectively. A negative result was assigned if no amplification occurred, if the CP value was equal to or exceeded 45/50. For conventional PCR a positive result was assigned if a PCR product of the correct size revealed the specific sequence. A result was designated negative if products of non-specific size compared to the positive control or no amplicon were detected.

After PCR assay validation, the workflow for PCR using aDNA started at the ArchaeoBioCenter using new loads and clean products from the producers only. All PCR tubes were sealed before entering laboratories of IMB. There was one exception for aliquots prepared with master mix to run positive controls which were strictly added at IMB only.

### Sample preparation and aDNA extraction

In this study we extracted the aDNA from teeth and one humerus testing three different extraction protocols (Table S1). Each tooth was cleaned using soft tissues soaked with a 1% NaOCl solution (Sigma-Aldrich, Munich, Germany) and subsequently washed twice in sterile water (Ampuwa^®^, Fresenius Medical Care, Germany). After exposing each side to ultra violet radiation for 15 min, leaving approximately ten centimeters between light source and sample, the teeth were ground to fine powder using a ZrO_2_-coated mill, type MM 2 (Retsch, Haan, Germany). The powder was transferred to a sterile tube and stored at -20°C until use.

Samples were extracted following the silica-based extraction protocol C developed by Yang et al. [32] and modified by Wiechmann et al. [33] using an initial amount of approximately 400 mg of tooth and bone powder, respectively. During the whole extraction procedure, safe-lock tubes (Biopur Quality, Eppendorf, Wesseling-Berzdorf, Germany) were used that are certified to be free of DNA. After having finished the protocol, an additional elution step was added to recover leftover DNA fragments: 50 µl of QIAquick^TM^ EB buffer (Qiagen, Hilden, Germany) were again pipetted onto the column, incubated for 10 min at 37 °C in a Thermo-Shaker TS-100 (Peqlab, Erlangen, Germany) and centrifuged at 16,000 x *g* for 1 min.

To ascertain the ideal extraction protocol that releases as much DNA as possible, teeth from five individuals (B2, MP17-I, MP26-I, MP59-I and MPS1-I) were treated following two further extraction protocols also based on the principle of binding DNA to a silica matrix. For all three extraction approaches, powder from the same tooth was used to gain comparable results.

The second DNA extraction protocol was carried out exactly as previously published by Rohland & Hofreiter [34,35], using an initial amount of approximately 400 mg of tooth powder.

The third extraction method consisted of a combination of protocols, on the basis of methods described by Rohland et al. [36,34], starting with 250 mg of tooth powder. Extraction solutions were prepared according to Rohland et al. [36]. 5 ml of extraction solution were added to each 250 mg of tooth powder and incubated overnight in the dark on a shaker. After centrifugation for 2 min at 5,000 x *g* the supernatant was transferred into 2.5 ml binding buffer. 100 µl of silica suspension were added and the pH was adjusted to 4.0 by adding 30% HCl. The samples were incubated with agitation in the dark for 3 h followed by a centrifugation step for 2 min at 5,000 x *g*. After discarding the supernatant, the silica pellet was resuspended in 1 ml of binding buffer. The buffer-silica suspension was transferred into a new tube and centrifuged for 1 min at 16,000 x *g*. The supernatant was completely removed with a pipette and the silica pellet was resuspended in 1 ml of washing buffer. Again, a centrifugation step for 1 min at 16,000 x *g* followed and the supernatant was removed. The washing step was repeated once again, then a centrifugation step for 1 min at 16000 x *g* was conducted, and the remaining liquid was removed. The silica was dried at room temperature for 15 min with open lid and afterwards resuspended in 50 µl of TE buffer. After incubating for 10 min and occasional shaking the sample was centrifuged for 2 min at 16,000 x *g*. The supernatant was transferred to a new tube.

For all extraction protocols, the elution step was repeated. Each eluate was stored at -20 °C until use.

### Testing aDNA extracts using validated assays

*Y. pestis* specific PCR assays were repeated up to four times with each aDNA sample that was generated by applying the extraction protocol described by Wiechmann & Grupe [33]. In case of a complete negative aDNA sample, the *HLA* specific assay was carried out. If the latter was positive, it was presumed that the sample did not contain *Y. pestis* DNA at a detectable limit. If it was negative, the aDNA sample was spiked with the synthetic *HLA* DNA to determine whether the extract was inhibited.

## Results

### Study collectives

To trace the plague agent *Y. pestis* we investigated aDNA extracts gained from 29 individuals from three different burial grounds located in the south (Manching-Pichl, Bavaria) and the north of Germany (Brandenburg), as well as in Basel, Switzerland.

**Figure 2:**
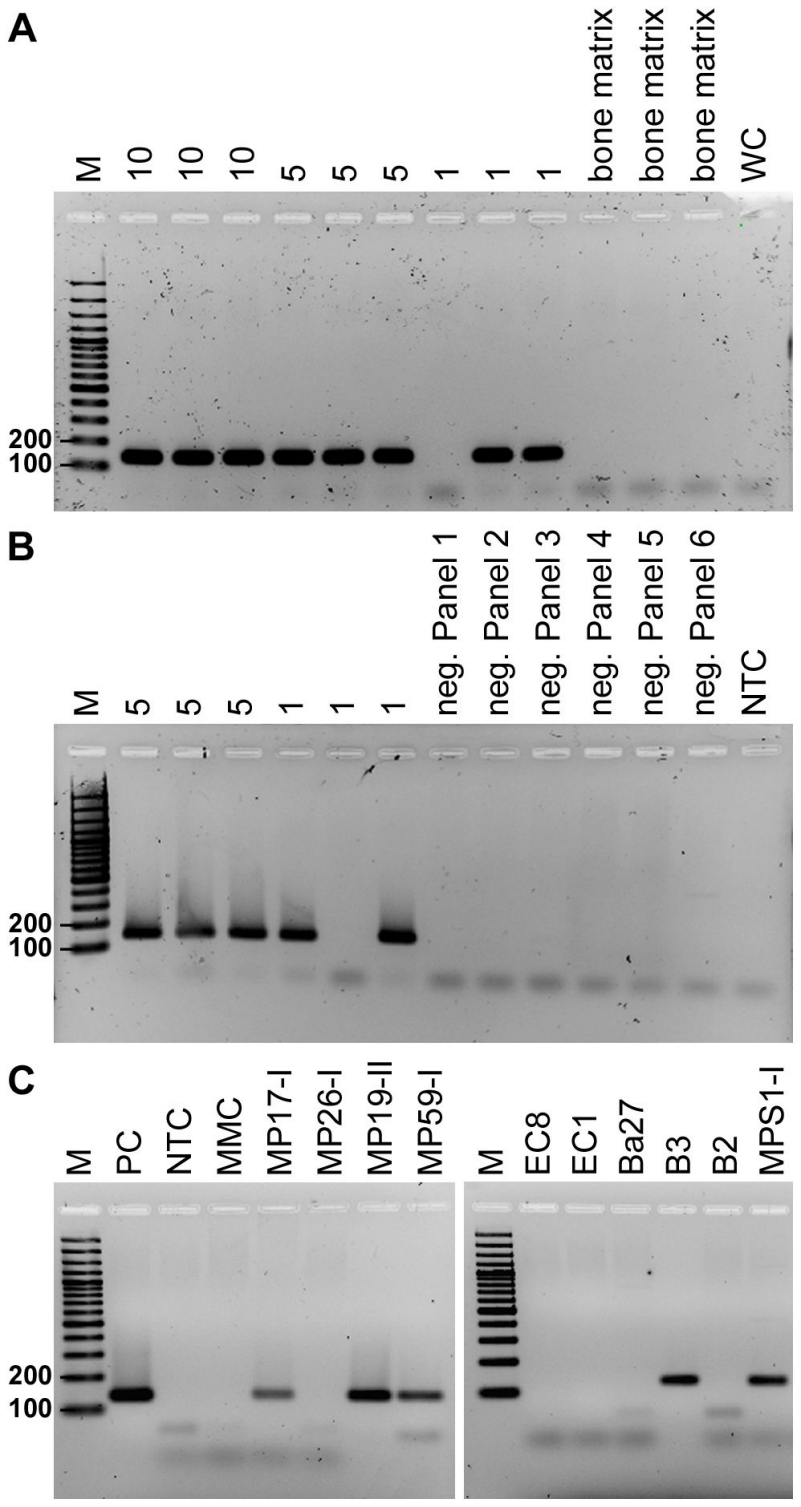
Amino acid profile of specimen MP 59-I, channel 1 (570 nm) and 2 (440 nm). The profile represents a typical collagenous pattern with a high amino acid yield. However, aspartic and glutamic acid concentrations are significantly reduced. At 72 min an unidentified peak was detected that did not show up in the other samples.

Initial collagen preservation analyses in three individuals from the Manching-Pichl mass grave yielded collagen net weight percentages of 2.4 (MP 54-II), 3.7 (MP 59-I), and 8.0 (MP 68-I). Regarding quality, C/N molar ratios were all within the range of 2.9 to 3.6, which is typically attributed to well preserved collagen [37,38]: 3.4 for MP 54-II, 3.4 for MP 59-I, and 3.2 for MP 68-I. The % C and % N values of the three individuals, MP 54-II (40% C, 14% N), MP 59-I (41% C, 14% N), and MP 68-I (39% C, 14% N) showed only a minor deviation compared to modern bone (35% C, 11-16% N) [38]. The only exception was the depletion of aspartic and glutamic acid in samples MP 59-I and MP 68-I, and a significant, unidentified peak after a retention time of 72 min in specimen MP 59-I (Figure 2). The overall amino acid profiles mostly displayed a pattern typical for collagen [25].

### Efficiency of the Quantitative Screening PCR

Testing different master mixes and titration of MgCl_2_ yielded differences of up to tenfold copy numbers in the detection limit of the artificial DNA standard. Adding BSA as an adsorbent for humic acids in aDNA revealed inhibitory effects starting at a concentration of 4 mg/ml. The assay revealed to be robust concerning various concentrations of primers and probe. Various annealing temperatures did not significantly influence the results. We therefore decided in favour of the following protocol in a total volume of 20 µl: 1x Platinum^®^ Quantitative SuperMix-UDG (Invitrogen/ Life technologies, Darmstadt, Germany), 6 mM MgCl_2_ (Applied Biosystems, Life technologies, Darmstadt, Germany), 0.4 mg/ml BSA (Ambion/ Life Technologies, Darmstadt, Germany), 0.25 µM of each primer (TibMolbiol, Berlin, Germany), 0.1 µM of the probe (TibMolbiol, Berlin, Germany), and 2.0 to 4.0 µl DNA. Cycling conditions started with an initial uracil cleaving step at 50 °C for 2 min, followed by a PCR activation step at 95 °C for 10 min. Then, 50 cycles were run at 95 °C for 15 sec and 60 °C for 30 sec. Final cooling was carried out at 40 °C for 30 sec.

Probit analysis revealed a detection limit of 3.62 copies with a probability of 95%. The linear range of the *pla* qPCR ranged from 10^4^ to 10 copies (Figure S1).

### Efficiency of confirmatory conventional PCR assays

**Figure 3:**
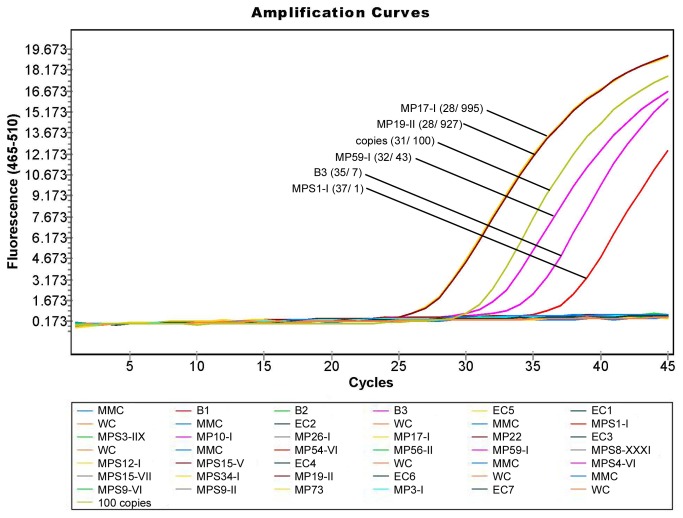
Conventional PCR targeting a 133 bp fragment of *Y. pestis pla* gene. A) Amplification products of the synthetic *pla* construct at concentrations of 10, 5 and 1 copy, respectively, are shown. Three samples were extracted from ancient bones originating from a modern time cemetery lacking any *Y. pestis* specific amplicons (bone matrix). B) Besides the synthetic *pla* construct, the gel shows products of PCR reactions set up, containing 1 ng DNA of each from the negative panel organisms (Table 2). C) Gel showing amplification products of aDNA extracted from teeth (Table 1). M: marker, PC: positive control, NTC: no template control, MMC: master mix control, WC: water control, MP: Manching-Pichl, Germany, origin of individuals, E1-8: extraction controls, Ba: Basel, Switzerland, origin of individuals, B: Brandenburg, Germany, origin of individuals.

Testing different Taq polymerases and master mixes revealed differences in the detection limit regarding the artificial target DNA. Also the production of non-specific amplicons – ladders – was observed in the ancient bone matrix using several Taq polymerases. A third issue was the reduction of amplicon concentration when using low template concentration. Awkwardly, the *Multiplex* PCR Master Mix (Qiagen, Hilden, Germany) revealed the best LOD by far and did not produce any smear, ladder or side product when processed with aDNA. Testing touch down cycling conditions with the Multiplex PCR Master Mix (Qiagen, Hilden, Germany) we even succeeded in amplifying single copies of target DNA (Figure 3). All other PCR parameters were robust and did not show significant changes in the results.

We therefore decided for the following protocol in a total volume of 50 µl: 1x Multiplex PCR Master Mix (Qiagen, Hilden, Germany), 0.01 U/µl UDG (Roche, Mannheim, Germany), 0.4 mg/ml BSA (Ambion/ Life technologies, Darmstadt, Germany) and 0.2 µM of each primer (TibMolbiol, Berlin, Germany). PCR cycling started with an uracil cleaving step at 25 °C for 10 min and a subsequent PCR activation step at 95 °C for 15 min for both assays (*pla* and *caf1*). For the *pla* specific assay further touchdown cycling started with 2 cycles at 94 °C for 30 sec, 64 °C for 30 sec and 72 °C at 60 sec, and 2 cycles at 94 °C for 30 sec, 62 °C for 30 sec and 72 °C at 60 sec. These two steps were followed by 46 cycles at 94 °C for 30 sec, 60 °C for 30 sec and 72 °C for 60 sec. For the *caf1* specific assay the cycling program continued with 50 cycles at 94 °C for 30 sec, 64 °C for 30 sec and 72 °C at 60 sec. Amplification ended with a final elongation at 72 °C for 10 min and cooling at 8 °C until analysis.

Characteristic amplicons of 133 bp (*pla*) and 161 bp (*caf1*) in length were visualized in a 2% agarose gel (Figure 3). Probit analysis revealed a detection limit of 4.24 copies with a probability of 95% (*pla*). Accuracy of all PCR assays revealed 100% regarding the specificity panel (Table 2, Figure 3).

### Absence of inhibitors

After the *HLA* assay validation we chose a final protocol in a total volume of 20 µl containing 1x Platinum® Quantitative PCR SuperMix-UDG (Invitrogen/ Life technologies, Darmstadt, Germany), 0.4 mg/ml of BSA (Ambion/ Life technologies, Darmstadt, Germany), 0.3 µM of each primer, 0.2 µM of probe, and 2 µl of template DNA. PCR cycling started with an uracil cleaving step at 50 °C for 2 min, and a subsequent PCR activation step at 95 °C for 10 min. 45 cycles were conducted at 95 °C for 15 sec and 60 °C for 30 sec. Final cooling was carried out at 40 °C for 30 sec. The probit analysis of *HLA* PCR revealed an efficiency of 12.19 copies with a probability of 95%.

### aDNA extraction

To evaluate the efficiency of the three extraction protocols the obtained CP values and accordant *pla* copy number were compared (Table 3). Applying the first method [33] three out of five samples contained detectable amounts of *Y. pestis* DNA. Method three [34,36] achieved slightly inferior results, detecting also three out of five samples but at a later CP. Finally method two [34,35] detected only two out of the five samples (Table 3).

**Table 3:** Comparison of the extraction method by qPCR.

**Individual**	**Method**	***pla* gene spec qPCR, mean of CP value**	**Calculated *pla* copy count per 1 µl**	**qPCR HLA geneCP value**
MP-17 I	1	28	460	-
	2	34	7	-
	3	29	210	-
MP26-I	1	neg	-	32
	2	neg	-	n.t.
	3	neg	-	35
MP59-I	1	32	22	
	2	neg	-	34
	3	36	2	-
MPS01-I	1	36	2	-
	2	>40	≤ 1	-
	3	37	1	-
B2	1	37*	≤ 1	-
	2	neg*	-	38
	3	neg*	-	34

* 4 µl instead of 2 µl of template aDNA were used

In two out of five tested individuals, B2 and MP26-I, initial *pla* amplification failed when using 2 µl of template DNA – regardless of the extraction method used. Using 4 µl of template DNA yielded one positive result for the extract from individual B2 extracted according to the first method (Table 3). Regardless of the extraction method, sample MP26-I did not contain specific DNA other than human DNA and was further regarded as a negative sample.

As proposed by Rohland & Hofreiter [34], we produced second eluates in each extraction round. Testing those by qPCR, we yielded a positive result for five individuals (Table S1).

### aDNA Testing using optimized extraction and validated PCR protocols

**Figure 4:**
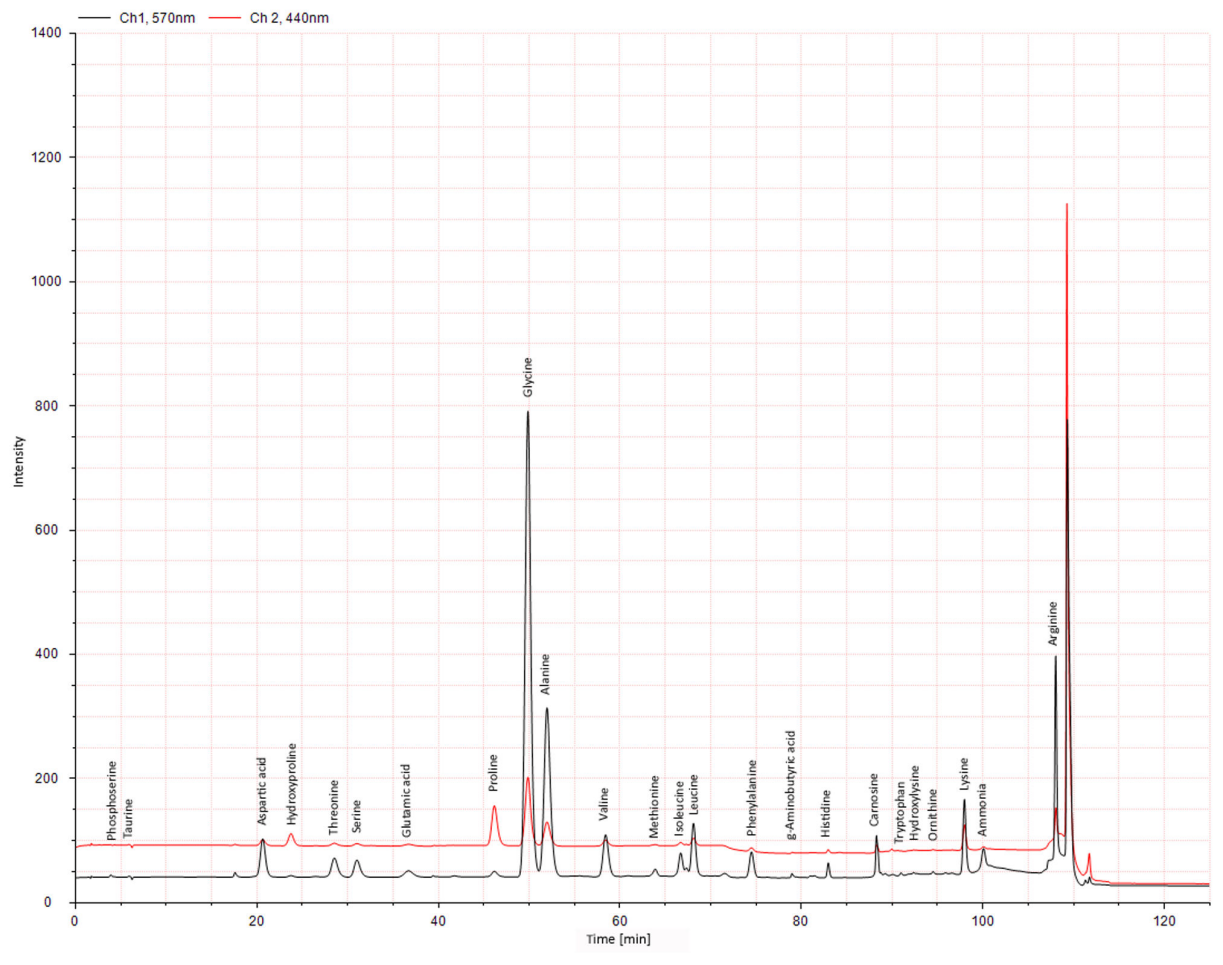
qPCR targeting *Y. pestis pla* gene screening ancient samples.

The final aDNA extraction was performed on 29 individuals applying the first described method [33] (Table 1). Seven of them, four from Manching-Pichl and three from Brandenburg, were tested positive for *Y. pestis* specific DNA in the qPCR screening assay (Figure 4, Table 1, S1). Regarding reproducibility, four of these samples resulted in four out of four positive results. Best results were obtained for sample MP19-II and MP17-I, with 1,400 and 1,120 *pla* copies in 2 µl sample volume, respectively (Table 1, S1).

 In five aDNA samples (MP17-I, MP19-II, MP59-I, MPS1-I, B3) a partial sequence of the *Y. pestis* specific *pla* gene could be amplified in qPCR as shown by amplification curves. Due to the positive standard of 100 copies of the artificial target gene in position H12, quantification could be carried out. Numbers in brackets represent rounded CP values and calculated *pla* copy numbers for each sample. None of the negative controls showed a signal: NTC: no template control, WC: water control, MMC: master mix control, EC2-7 extraction controls.

Testing the 29 individuals confirmatory by conventional PCR assays and sequencing resulted in specific *pla* gene sequences for five individuals, and specific *caf1* gene sequences for four individuals. The result was repeatedly obtained in four (*pla*) and three cases (*caf1*) (Table 1, S2). All sequences revealed up to 100% identity to the correspondent reference sequences (GenBank accession number AL109969/AL117211.1). Despite UDG application, one C◊T DNA transition typical for aDNA was observed in two amplicons of two samples (Figure S2). However UDG does not cleave 100% of uracil bases in a sample. For sample MP17-I we received a correct sequences regarding further amplicons. This was not the case for sample MPS1-I, which contained less *Y. pestis* specific DNA. And the application of UDG might have even cleaved possible remaining DNA (Table 1,3; Figure S1).

DNA extracts from 22 individuals, including all from Basel, Switzerland, failed in all repeatedly attempted *Y. pestis* specific *pla* PCR assays (Table 1, S1). They were then tested in the *HLA* specific qPCR assay to determine inhibition. Out of 22, 12 individuals yielded a positive *HLA* result. The DNA of the ten other individuals was tested negative and therefore spiked with 100 copies of the artificial *HLA* construct. All of the spiked samples revealed a positive result, demonstrating the absence of any PCR inhibitor.

All extraction controls generated throughout the workflow were tested negative in any specific assay.

## Discussion

Detection of pathogen specific DNA in ancient samples such as skeletons is crucial and requires validated detection assays in order to avoid false positive or false negative results. Therefore, one major goal of the study focused on the testing of medieval human samples using a similar diagnostic approach as recommended for the detection of highly pathogenic bacteria such as *Y. pestis* in clinical human samples of recent plague victims [39]. Although historical samples are no longer infectious, a plague case definition is nevertheless important for historical and epidemiological reasons, as answers about its pandemic character and virulence still need to be found. Upon detection of *Y. pestis* specific DNA in the ancient tooth pulp, it can be assumed that the clinical picture of septicemic plague had been existent and was the most probable cause of death to the human individual [39]. Nowadays, plague diagnostics takes place in an accredited laboratory, such as the Malagasy WHO Collaboration Center for Plague, a facility of the American Laboratory Response Networks (LRN), or the IMB’s German ISO 15189 certified central diagnostic laboratory. To get to this status, certain requirements need to be fulfilled and are controlled regularly.

Retrospective diagnostics - here dating back at least 500 years - only use DNA detection methods leading to preliminary results, which need to be affirmed by alternate or more extensive approaches. Also PCR with aDNA will often require much more optimization than PCR with modern DNA because template quantity, quality, and level of inhibition are unique to each extract [17]. In a recent study, more than 1000 tooth pulp specimens were screened by high throughput detection qPCR and none of the samples turned out positive [19]. However, assays were not validated, and no LOD was determined. As a consequence it is not justified to conclude that pathogen specific DNA was absent in the samples.

In the present study we used methods carefully validated according to ISO 15189 [40]. We followed an algorithm starting with a screening and quantification PCR (*pla*) and then confirmed positive samples using amplicon sequencing (*pla*) and an additional target within the *Y. pestis* genome (*caf1*). Our study clearly resulted in precise analysis of *Y. pestis* DNA content for each of the investigated samples.

A second major challenge in this study was to follow a strategy that combines the workflow of a modern diagnostic laboratory and obeying rules for authenticity during the work with aDNA at the same time (Table 4). Extracts are usually characterized by a low number of endogenous molecules that are fragmented and chemically altered. Also exogenous sources of contamination, and finally false negative results have to be considered [16,41,42]. Since more than ten years criteria of authenticity have been published by different aDNA working groups (Table 4) [14–16], being subject to controversial discussion today [43]. Following Gilbert et al. who suggests the critical consideration of all available information instead of asking which criteria can be checked off a list [44] we used the published criteria as a discussion guideline (Table 4).

**Table 4:** Criteria for authenticity.

***Recommendedcriteriaforauthenticity(accordingto[14–16***])	**Strategies applied in this study**
Physically isolated pre-PCR-facility with strict decontamination strategy in place using bleach and UV-light, movement only from pre-PCR to post-PCR area	Use of an aDNA laboratory completely isolated and located in a different building than all post-PCR laboratories (Figure 1); routine application of a strict one way regime (Figure 1.) and decontamination strategies for samples, surfaces, reagents and tools in place
Extraction and PCR controls	Blank controls were performed for each set of extracts or PCR round to a ratio of 1:7 or 1:8
Reproducibility	Multiple PCR rounds from the same or different extractions of each individual yielded consistent results (Table S1)
Quantification of starting templates	Quantification of the number of starting molecules was carried out by quantitative real time PCR but mainly for methodological reasons and less to prove authenticity
Appropriate molecule behaviour	We observed an inverse correlation between amplification efficiency and length of amplicon (Table 1)
UDG treatment (only [16])	UDG treatment was carried out prior to each PCR round
Cloning	Direct sequencing of all PCR products was sufficient in this study, cloning was not attempted
Independent replication in another aDNA laboratory	In the present study an independent replication was considered not to be necessary; however, the results of a previous study [9] can be considered as independent replication because the previous study had been carried out in a different laboratory and by different personnel
Biochemical preservation	The biochemical preservation of bone collagen analysed exemplarily for three individuals indicated that at least the specimens of the largest of the three archaeological sites were preserved well enough to allow DNA analysis
Preservation of associated remains	No associated remains e.g. of animals were excavated; instead, human host DNA was amplified, but the significance of the results is limited

Below, three different types of contamination qualities are discussed. Type 1 is the pre-laboratory contamination during burial, excavation, or handling of the skeletal remains by researchers before introducing the samples into an aDNA laboratory. Type 2 is an intra-laboratory contamination caused by modern DNA, e.g. due to the use of contaminated plastic ware and reagents or due to handling modern DNA sources. Finally, type 3 contamination is caused by cross-contamination with amplicons from previously run PCRs.

Contaminations of type 1 can be attributed to microorganisms from the burial site, particularly when universal, non-specific assays for bacterial DNA are used [18]. We can exclude this issue in our study for several reasons: we used two *Y. pestis* specific molecular targets, *pla* and *caf1* [2,29,45]. However, parts of the *pla* gene were recently found to exist in the rat genome [46]. *Y. pestis* is not a soil bacterium, and is not endemic in Germany these days [3]; we nevertheless removed potential surface DNA contamination as explained above. Finally, 16 of the investigated individuals from the mass grave Manching-Pichl as well as all individuals from Basel, Switzerland, remained negative for specific targets of *Y. pestis* in all assays, although some of them were buried side by side with positive individuals (Table 1, S1).

To prevent type 2 and 3 contaminations, we ran a strict one way regime and worked in separated laboratory complexes (Figure 1, Table 4). Although spatial separation is recognized to be essential for aDNA studies, it has not been applied very often in studies of ancient pathogens (Review in [47]), leading to justifiable skepticism about the validity of certain studies on ancient *Y. pestis* DNA [18].

To detect any sporadic contamination, we routinely used ubiquitous controls as previously demanded (Table 4) [16,41]. However, false positive results are still difficult to detect, some argue that negative blank controls may easily conceal low-level laboratory contamination, owing to a poorly understood carrier effect [16,48]. But, in the present study all controls as well as the above mentioned 22 ancient samples remained negative during repeated testing (Table 1, S1; Figures 3, 4).

Sporadic, non-reproducible positive signals for the samples B1 and B2 were achieved by the quantification assay targeting *pla* (Table 1, S1). The determined quantities of 1-3 *pla* gene copies match the LOD and therefore the non-reproducible positive results are perfectly explained.

Cooper and Poinar recommended that “the copy number of DNA target” should be assessed and “when the number of starting template is low (< 1000) it may be obnoxious to exclude the possibility of sporadic contamination” [14], while Pääbo et al. stated that for extracts containing > 1000 molecules a single assay repetition is sufficient, and only if fewer molecules are present, several assay repetitions are needed (Table 4) [41]. This criterion is disputed and not widely used [43,47]. We decided to repeat the assays anyway three to four times with each extract and performed quantitative PCR (Table 1, S1). Both, appropriate molecule behavior as well as repeated amplification and quantification of target DNA underline the authenticity of our results (Table 4). We observed an inverse correlation between amplification success and amplicon length. As shown in Table 1, it was possible to amplify the 70 bp fragment (*pla*) in qPCR more often than the 133 bp fragment (*pla*), and the 161 bp fragment (*caf1*) regarding individuals MPS01-I, B1 and B2.

Repeated amplification and sequencing from different extracts can also serve to detect false sequencing results due to damage induced errors (Table 4, Figure S2). The main type of diagenetic DNA damage is deamination of cytosine converting it to uracil, which produces sequencing errors [49,50]. All of our PCR assays included UDG to cleave the aDNA strand at most altered positions. In twelve *pla* sequences and five *caf1* sequences we detected two single C>T transitions (MP17-I, MPS1-I in Figure S2). We favored the use of UDG and accepted a consequential loss of aDNA molecules, even risking complete negative results for certain samples. However, without the application of UDG the generated sequences might have included numerous sequencing errors that would have needed revision [51,52]. Furthermore we agree with others in the field, that DNA damage can also be caused during handling and processing [34,53], and therefore decided not to use the assessment of DNA damage as criterion for authenticity.

DNA preservation as valuated by several techniques and protein preservation seem to be correlated [54–58]. In our study, aDNA preservation status and the quality of bone collagen from three individuals from the mass grave in Manching-Pichl also correlate (Figures 2-4, Tables 1, 4, S1). Amino acid analysis of ancient organic material is a powerful tool to identify compositional changes within a polypeptide, thus providing key information on the macromolecular preservation of bone collagen [25].

To prevent false negative results due to damaged DNA or heterogeneity in the amplification products resulting from contamination, cloning of amplification products has often been recommended (Table 4) [14,16,41]. However, cloning poses other major sources of contamination [51]. Due to unambiguous amplicon sequencing, cloning was not part of our study.

The independent replication of results regarding aDNA studies by different working groups is recommended (Table 4). However, we agree with other aDNA experts that it is only necessary if novel or unexpected results were obtained [41,43], which was not the case in our study. Furthermore, *Y. pestis* specific DNA has already been detected in some individuals from Manching-Pichl in a different laboratory [9].

To optimize DNA yield, we compared different DNA extraction methods. After quantifying *Y. pestis* specific DNA we chose the method yielding positive results for one further individual (B2) (Table 3) [33]. Here, the extraction solution additionally contained SDS. As a strong detergent it supports DNA release, but might also decrease DNA quality and can act as PCR inhibitor [34]. Also incubation temperature was higher during this extraction, a factor responsible for kinetics and efficiency of enzymes such as proteinase K [34]. To attain more DNA, we repeated the final elution step, although the second or even further eluates showed higher CP values (Table S1). The finally selected extraction protocol also included the least number of steps and the use of an easy to handle kit. Therefore, the general contamination risk was even more reduced.

Following a diagnostic algorithm we used an initial screening and quantification assay which was confirmed by conventional PCR and sequence analysis targeting two different virulence plasmids specific for the *Y. pestis* genome. All assays were meticulously validated according to ISO 15189 requirements regarding efficiency, sensitivity, specificity, LOD and purity of amplicons with respect to non-specific products (Table 2, Figure 2, S1) [40].

Both target genes, *pla* and *caf1*, located on plasmids pPCP1 and pMT1 are specific for *Y. pestis* and do not even occur in the closest relatives *Y. pseudotuberculosis* or *Y. enterocolitica* [2,29,45]. They have successfully been employed in the detection of the pathogen in aDNA [8–12,33,59] and modern clinical samples before [60].

The approach described by Schuenemann et al. [11] provided single- and multiplex assays for the detection of the *pla* gene and its flanking intergenic spacers using intercalating SYBR fluorescent technique with the same detection limit of 4 gene copies as presented in this study. The reported maximum copy number of *Y. pestis* specific DNA was 30 copies/µl [11]. The maximum of calculated copy numbers in our study was 700 copies/µl. Despite the use of UDG we exceeded the efficiency of the previously published qPCR assay by more than 20-fold [11] although we are aware that we cannot directly compare the two studies, as the ancient material originated from different sources. Besides, SYBR green assays are less specific than assays with homologous binding fluorescent probes as used in our study, and therefore need further confirmation by cloning and sequencing. Nguyen-Hieu et al. published a qPCR assay using binding fluorescent probes for the detection of *Y. pestis* and six other pathogens [19]. They screened more than 1000 samples. However, none was positive for *Y. pestis*. As there was no LOD determined it is not possible to explain a negative result. In our study we went one step further, applying probes with locked nucleic acids (LNA). LNA bases change the conformation of the DNA helix, increase the stability and the melting temperature of the duplex [61]. Specificity is increased at the same time as the target size can be reduced. Except for the SYBR green singleplex assay discussed above [11], our qPCR assay targets the shortest *pla* fragment (70 bp), contrary to previously published PCR assays [8–10,12,19,33,59]. 70 bp often constitutes the upper limit for successful aDNA detection [62–64]. Comparing the amplification success of our qPCR assay to conventional PCR, both having a LOD of four gene copies, the qPCR still detected two more samples out of seven and had a better overall detection rate regarding extracts with less than 44 *pla* copies (Table 1, S1).

## Conclusions

We describe a strategy for retrospective diagnostics regarding medieval human plague victims based upon thoroughly validated PCR assays. 29 individuals originating from three different burial sites were initially regarded as potential victims of the Black Death pandemic. 7 samples (24%) were positive in the *Y. pestis* specific *pla* gene screening qPCR assay. Confirmation through second target *caf1* specific PCR was successful for 4 of the *pla* positive individuals (14%). A maximum of 700 and 560 copies per µl aDNA were quantified in two Manching-Pichl samples. They were positive in all *Y. pestis* specific assays including all repetitions, and are thus candidates for future continuative investigations such as whole genome sequencing. We further conclude that the precautions taken for the work with aDNA in this study are sufficient to prevent external sample contamination and fulfill the criteria of authenticity. With regard to the uniqueness of ancient material and the numerous debates about authenticity of results we strongly recommend using a careful strategy and validated assays as presented in our study.

## Supporting Information

Table S1 **CP values and PCR results of all investigated samples.**
(XLSX)

Figure S1 **Linearity of the qPCR targeting *Y. pestis* specific *pla* gene.** In a threefold repetition of each dilution the linearity of the assay was determined. The assay is linear in the tested range of 10 E4 to 10 E1 copy.
(TIF)

Figure S2 **Alignment of partial *pla* and *caf1* specific sequences.** Despite the use of UDG, two CT transitions are present in aDNA sequences targeting the genes *pla* and *caf1* that were aligned to the reference sequences (AL109969/AL117211). Those errors result from deamination of cytosine and are typical for aDNA.
(PDF)
