# Correction: Strategy for Sensitive and Specific Detection of *Yersinia pestis* in Skeletons of the Black Death Pandemic

**DOI:** 10.1371/annotation/190eceb9-03c6-4d5f-b861-83d44f2a0e53

**Published:** 2013-10-21

**Authors:** Lisa Seifert, Michaela Harbeck, Astrid Thomas, Nadja Hoke, Lothar Zöller, Ingrid Wiechmann, Gisela Grupe, Holger C. Scholz, Julia M. Riehm

The titles and legends for Figures 1-3 were incorrectly switched. The title and legend currently appearing with Figure 1 belong with Figure 3, the title and legend currently appearing with Figure 2 belong with Figure 1, and the title and legend currently appearing with Figure 3 belong with Figure 2. The Figures themselves are in correct order. 

