# Supplementary figures and images for: Strategy for Sensitive and Specific Detection of Yersinia pestis in Skeletons of the Black Death Pandemic
Source: PLoS One. 2013 Sep 17;8(9):e75742. doi: 10.1371/journal.pone.0075742 (PMC3775804; doi:10.1371/journal.pone.0075742)

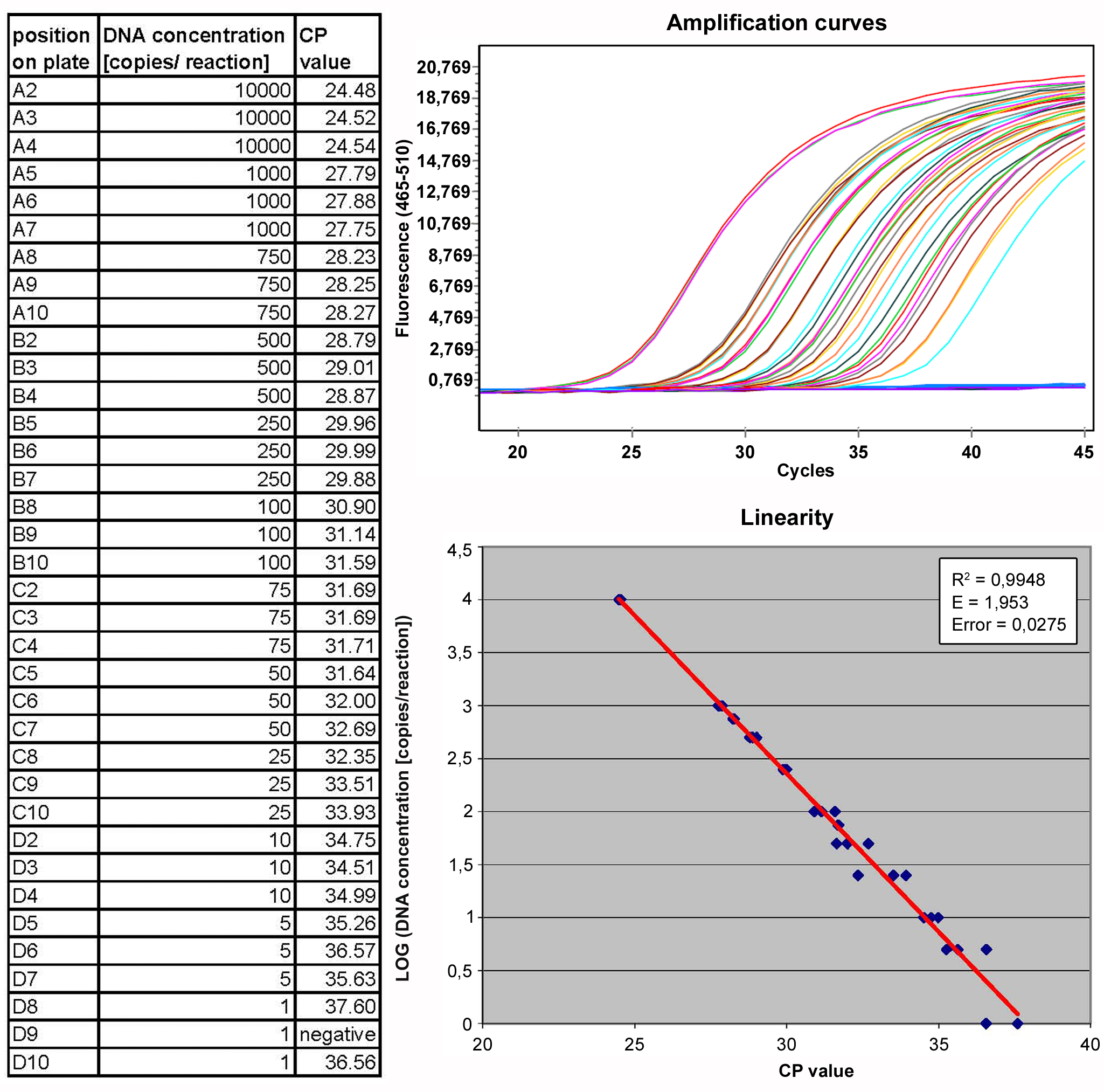

Supplement: Figure S1 — Linearity of the qPCR targeting Y. pestis specific pla gene. In a threefold repetition of each dilution the linearity of the assay was determined. The assay is linear in the tested range of 10 E4 to 10 E1 copy. (TIF) [file pone.0075742.s002.tif]
