# Supplementary material for: Strategy for Sensitive and Specific Detection of Yersinia pestis in Skeletons of the Black Death Pandemic
Source: PLoS One. 2013 Sep 17;8(9):e75742. doi: 10.1371/journal.pone.0075742 (PMC3775804; doi:10.1371/journal.pone.0075742)
Supplement: Figure S2 — Alignment of partial pla and caf1 specific sequences. Despite the use of UDG, two CT transitions are present in aDNA sequences targeting the genes pla and caf1 that were aligned to the reference sequences (AL109969/AL117211). Those errors result from deamination of cytosine and are typical for aDNA. (PDF) [file pone.0075742.s003.pdf]

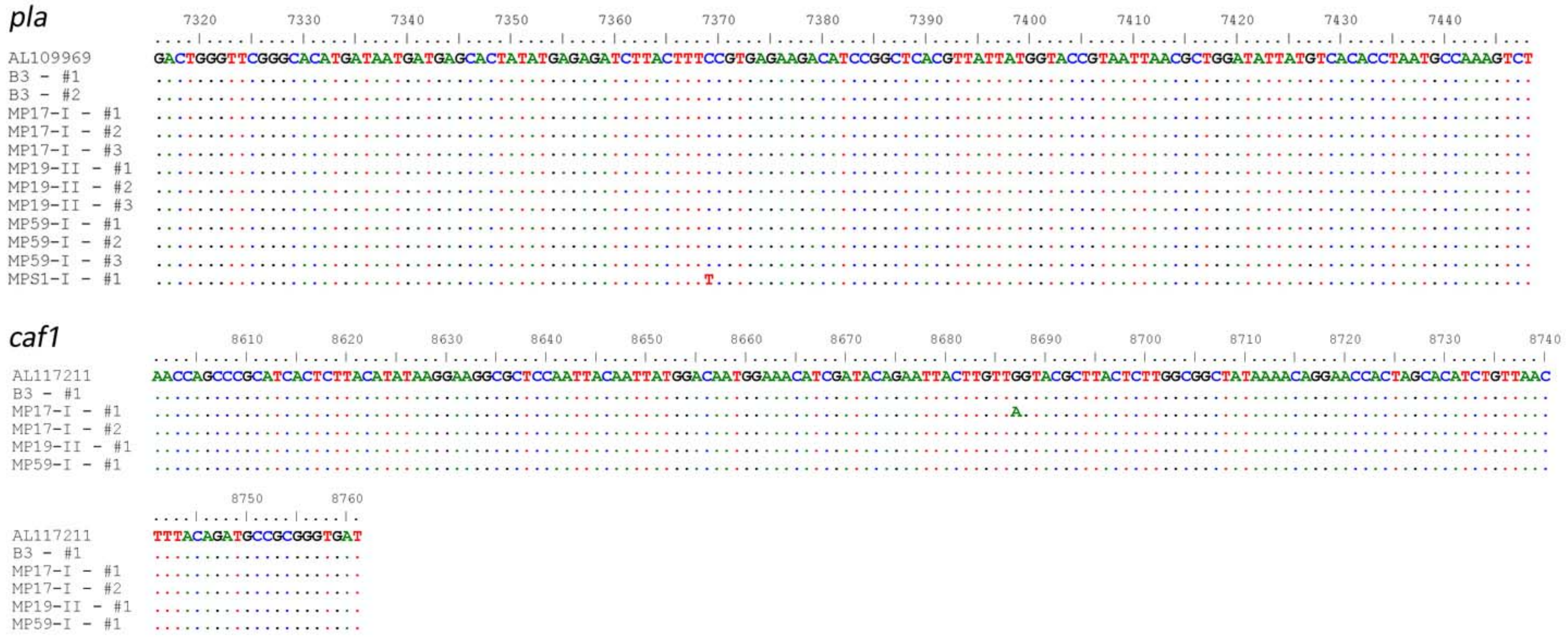

pla
7320
7330
7340
7350
7360
7370
7380
7390
7400
7410
7420
7430
7440
AL109969
GACTGGGTTCGGGCACATGATAATGATGAGCACTATATGAGAGATCTTACTTTCCGTGAGAAGACATCCGGCTCACGTTATTATGGTACCGTAATTAACGCTGGATATTATGTCACACCTAATGCCAAAGTCT
B3 - #1
B3 - #2
MP17-I - #1
MP17-I - #2
MP17-I - #3
MP19-II - #1
MP19-II - #2
MP19-II - #3
MP59-I - #1
MP59-I - #2
MP59-I - #3
MPS1-I - #1
T
caf1
8610
8620
8630
8640
8650
8660
8670
8680
8690
8700
8710
8720
8730
8740
AL117211
AACCAGCCCGCATCACTCTTACATATAAGGAAGGCGCTCCAATTACAATTATGGACAATGGAAACATCGATACAGAATTACTTGTTGGTACGCTTACTCTTGGCGGCTATAAAACAGGAACCACTAGCACATCTGTTAAC
B3 - #1
MP17-I - #1
A
MP17-I - #2
MP19-II - #1
MP59-I - #1
8750
8760
AL117211
TTTACAGATGCCGCGGGTGAT
B3 - #1
MP17-I - #1
MP17-I - #2
MP19-II - #1
MP59-I - #1
